# Functional variation of SLC52A3 rs13042395 predicts survival of Chinese gastric cancer patients

**DOI:** 10.1111/jcmm.15798

**Published:** 2020-09-05

**Authors:** Xiaofei Qu, Lei Cheng, Liqin Zhao, Lixin Qiu, Weijian Guo

**Affiliations:** ^1^ Department of Oncology Shanghai Medical College Fudan University Shanghai China; ^2^ Cancer Institute Collaborative Innovation Center for Cancer Medicine Fudan University Shanghai Cancer Center Fudan University Shanghai China; ^3^ Department of Medical Oncology Fudan University Shanghai Cancer Center Shanghai China

**Keywords:** gastric cancer, prognosis, single nucleotide polymorphism, SLC52A3

## Abstract

The solute carrier family 52 member 3 (SLC52A3) gene encodes riboflavin transporter protein which is essential to maintain mitochondrial function in cells. In our research, we found that *SLC52A3* rs13042395 C > T variation was significantly associated with poor survival in a 926 Chinese gastric cancer (GCa) patients cohort (CC/CT genotype versus TT genotype, HR = 0.57, 95%CI (0.40‐0.82), log‐rank *P* = 0.015). The *SLC52A3* rs13042395 C > T change led to its increased mRNA expression according to expression quantitative trait loci analysis (*P* = 0.0029). In vitro, it was revealed that rs13042395 C allele had higher binding affinity to inhibitory transcription factor Meis homeobox 1 (MEIS1) compared with T allele, knock‐down of *MEIS1* could up‐regulate *SLC52A3*, and overexpression of *SLC52A3* contributed to the increased ability of proliferation, colony formation, migration and invasion in GCa cells. Subsequently, the bioinformatics analysis combined with experiments in vitro suggested that Gap junction protein alpha 1 (GJA1) was the downstream effector of *SLC52A3*, *SLC52A3* may promote the GCa cells aggressiveness by down‐regulating the *GJA1* expression. Overall, *SLC52A3* genetic variant rs13042395 C > T change was associated with poorer survival in Chinese GCa patients and increased *SLC52A3* expression by interaction with *MEIS1. SLC52A3* promoted the GCa cells aggressiveness by down‐regulating the *GJA1* expression.

## 1 INTRODUCTION

Over 679 000 patients suffered from gastric carcinoma (GCa) with an estimated nearly 500 000 GCa‐related deaths in 2015, making it the second most deadly cancer in China.^1^ Despite the advance achieved in surgery, chemotherapy, radiotherapy and targeted therapies in the past few decades, the median overall survival (OS) of advanced GCa remained 10‐12 months.^2^ Hence, it was critical to select poor survival GCa patients for earlier intervention which was promising to prolong the survival time of them.

Recently, several biomarkers were identified for the prognosis of GCa which was potentially able to select high‐risk patients, such as circulating non‐coding RNA and some somatic genes mutations.^3^, ^4^ Several germline mutations have already been investigated for the prognosis in GCa.^5^, ^6^ However, few of them further explored the molecular mechanism underlying the prognosis effect.

The solute carrier family 52 member 3 (SLC52A3) gene encodes the riboflavin transporter protein, which was crucial for the riboflavin absorption.^7^ Previous studies suggested that SLC52A3 gene was highly abnormally expressed in oesophageal tumour tissue, and the depletion of SLC52A3 gene would significantly inhibit the proliferation of oesophageal carcinoma cells,^8^ which indicated that SLC52A3 gene played an important role in the progression of cancer cells. Germline mutation (rs13042395 C > T) on SLC52A3 gene was identified as a risk locus for gastric cancer incidence by genome‐wide association studies, and it was found that rs13042395 C > T change significantly increased the risk of cancer incidence in various cancer types, notably in the GCa patients.^9^, ^10^ However, the molecular mechanism remained ambiguous and the research on the association of germline mutation in the SLC52A3 gene and the survival of GCa patients was hardly seen. In the present study, we investigated associations between genetic mutations of the *SLC52A3* rs13042395 C > T change and survival of Chinese GCa patients and subsequently explored mechanistic basis of the observed associations.

## 2 METHOD AND MATERIAL

### 2.1 Study population

From January 2009 to March 2011, a total of 926 unrelated Han ethnic Chinese patients were recruited from Fudan University Shanghai Cancer Center (FUSCC) in Eastern China who was newly diagnosed or pathologically confirmed primary GCa. Peripheral blood samples of this GCa patients were provided by the tissue bank of FUSCC. All of the 926 patients had signed a written informed consent to donate their biological samples to the tissue bank for scientific research. Clinical information of each patients was collected. Our research proposal was approved by the FUSCC institutional review board.

### 2.2 Genotyping, quality control and survival analysis

DNA of study patients was extracted from peripheral blood cells. Single nucleotide polymorphisms (SNPs) were genotyped via a matrix‐assisted laser desorption/ionization time‐of‐flight (MALDI‐TOF) mass spectrometer using the MassARRAY Analyzer 4 platform (Sequenom, CA, USA). All the primers were designed by Assay Design Suite v2.0 from Mysequenom online software (www.mysequenom.com). The standard polymerase chain reaction (PCR) reaction was conducted in a total volume of 5 μL system containing 10 ng of genomic DNA. One negative control and one duplicate sample were used for quality controls in every 96‐well plates. Genotyping results of 5% patients were repeated, and the consistency was 100%.

A total of 926 patients were divided into two groups according to their rs13042395 genotype. Kaplan‐Meier (KM) curve was used to illustrate the difference and log‐rank test was performed to provide the statistics. Multivariate analysis was calculated by Cox proportional hazards regression which was adjusted by clinical variables. Stratification analysis was conducted to explore the interaction of the genetic variant with clinical variables.

### 2.3 Expression quantitative trait loci analysis and validation in vitro

Expression quantitative trait loci analysis was conducted using rs13042395 genotype and *SLC52A3* mRNA expression data in normal gastric tissue from the Genotype‐Tissue Expression (GTEx) database. The different expression violin plot was directly available from the GTEx website (https://www.gtexportal.org/).

The Jaspar web server (http://jaspar.genereg.net/) was used to detect the putative transcription factors binding to the *SLC52A3* rs13042395 allele. Subsequently, the binding affinity change resulted from rs13042395 C > T change was suggested. The Meis homeobox 1 (MEIS1) was found with the most significant binding affinity change, and *MEIS1* then was selected for the further research.

The association of *MEIS1* and *SLC52A3* expression was assessed by GEPIA webserver (http://gepia.cancer‐pku.cn), and plot figure was obtained. Validation experiments were performed. Three types of siRNA targeting the different site of *MEIS1* were purchased from the GenePharma (Shanghai China). These siRNA was used to transfect the BGC823 cells, respectively, using Lipofectamine™ RNAiMAX (Thermo Fisher, MA, Waltham, USA). siMEIS1‐1 sequence: CUGUCAAUGACGCUUUAAATT (forward); siMEIS1‐2 sequence: GCUCGUCAGAGUCAUUCAATT (forward); siMEIS1‐3 sequence: GCCUAUCGAUUUGGUGAUATT (forward). Western blotting and real‐time PCR were performed to detect the *MEIS1* and *SLC52A3* expression change.

### 2.4 Target sequencing and electrophoresis mobility shift assay

We sequenced the rs13042395 locus in BGC823, MGC803 and AGS cell lines using Sanger sequencing method to make sure these cell lines used in our research were rs13042395 CC genotype.

Electrophoresis mobility shift assay (EMSA) analysis was performed at Viagene Co. Ltd (Shanghai, China) and was used to detect the different binding affinities of nucleoprotein with the rs13042395 C or T allele flanked by 58bp DNA up‐ and downstream. The DNA sequence of rs13042395 C allele flanked by 58 bp nucleotide was as follows: 5′‐TGGGGTTCTGACCAGGGCCAGTGCACCGT**C**ATTGTGTGGGCTGGGCCATCTCCTCCAGG‐3’. The rs13042395 T allele flanked by 58 bp nucleotide was as follows: 5’‐TGGGGTTCTGACCAGGGCCAGTGCACCGT**T**ATTGTGTGGGCTGGGCCATCTCCTCCAGG‐3’. Bold characters in the sequence represent the rs13042395 locus. The probe representing the rs13042395 C allele (bio‐CLwild) was as follow: 5’‐CCAGTGCACCGT**C**ATTGTGTGGGCT‐3’, and the probe representing the rs13042395 T allele (bio‐CLmut) was as follow: 5’‐CCAGTGCACCGT**T**ATTGTGTGGGCT‐3’. The positive control probe was as follow: 5’‐CCAGTGCAAAGAGCTTGTGTGGGCT‐3’, and the negative control probe was as follow: 5’‐CGTACGCGCTGTCATATTGACAGGT‐3’. The nucleoprotein of BGC823 and AGS cell lines were extracted and incubated with different probes which were labelled with biotin. After that, nucleoprotein‐probes complex were separated by electrophoresis and then biotin signals were detected to assess the quantities of nucleoprotein‐probes complex.

In the competitive EMSA assay, the probe representing the rs13042395 C allele, which was not labelled with biotin was called CLwild. The standard probe binding MEIS1 protein (std‐ME1S1) was as follow: 5’‐CCAGTGCACTGTCATTGTGTGGGCT‐3’. The probe with modifications within the key motif binding MEIS1 (pc‐MEIS1) was as follow: 5’‐ CCAGTGCAAAGAGCTTGTGTGGGCT ‐3’. The probe with modifications outside the key motif binding MEIS1 (nc‐MEIS1) was as follow: 5’‐ CGTACGCGCTGTCATATTGACAGGT ‐3’. All the four above probes were not labelled with biotin and were, respectively, added into bio‐CLwild probes and BGC823 cell nucleoprotein to competitively bind to the transcription factors, assumedly the *MEIS1*.

In the super‐shift EMSA assay, *MEIS1* antibodies were added to ascertain whether the DNA‐protein complex can bind the antibodies.

### 2.5 Cell lines and culture

Human gastric cancer lines BGC823, MGC803 and AGS were obtained from the Cell Bank of Type Culture Collection of Chinese Academy of Sciences (Shanghai, China). All of the above cell lines have been authenticated by short tandem repeat (STR) DNA profiling analysis. Cells were cultured in RPMI 1640 medium supplemented with 10% FBS (Gibco, USA) and antibiotics at 37°C, 5% CO_2_.

### 2.6 Gene sets enrichment analysis, lentiviruses and infection

The gene sets enrichment analysis (GSEA) of two Gene Expression Omnibus (GEO) data sets (GSE62254 and GSE15459) and TCGA gastric cancer data set were conducted to calculated the most likely downstream effector gene of SLC52A3.

Lentiviral vectors expressing *SLC52A3* and Gap junction protein alpha 1 (GJA1) were obtained from Shanghai Hanyin Biotechnology Co Ltd. MGC803 and AGS cells were infected with lentivirus carrying *SLC52A3* or *GJA1*, and transfected cells were selected with indicated antibiotics to generate overexpressing stable cells. Western blotting was applied to detect the expression level of *SLC52A3* and *GJA1*, and *GAPDH* was used as the internal reference protein.

### 2.7 Western blotting and PCR

Firstly, the whole cell lysates were obtained as the supernatant, and protein concentration was determined using the Pierce BCA Assay Kit (Thermo Scientific). Then, a total of 30 μg protein was loaded into each lane and separated by a 10% SDS‐PAGE gel. After that, proteins were transferred into PVDF membranes. The membranes were blocked with 5% of skim milk for one hour at room temperature and incubated in primary antibodies at 4°C overnight. The next day, the membranes were washed with TBST and incubated with HRP‐conjugated secondary antibody for one hour at room temperature. Finally, the protein bands were detected with enhanced chemiluminescence (Millipore, Burlington, MA, USA) by a luminescent image analyser (ImageQuant LAS4000). Primary antibodies against *MEIS1* were purchased from Active Motif (Carlsbad, California, USA), *SLC52A3* and *GJA1* were purchased from Abcam (Cambridge, UK), primary antibody against GAPDH and the secondary antibodies were from Proteintech (Wuhan, China).

RNA was extracted using TRIzol reagent (Invitrogen, Carlsbad, CA, USA). A PrimeScript reagent Kit with gDNA Eraser (Takara, Dalian, China) was used to reverse transcribe RNA into cDNA, and then, quantitative real‐time PCR was performed with TB Green Ex TaqTM (Takara) using Applied Biosystems Prism 7900 system (Life Technologies, Carlsbad, CA, USA). The sequences of the primers were as follows: MEIS1:5’‐CTTCCCTCTCTTAGCACTGATT‐3’; SLC52A3: 5’‐GCATCGCCTTCTTGGTCCTCAC‐3’; GAPDH: 5’‐ACCCAGAAGACTGTGGATGG‐3’.

### 2.8 Proliferation, migration and invasion assay

The EdU cell proliferation assay and plate colony formation assay were used to evaluate the ability of cell proliferation. For EdU cell proliferation assay, cells were seeded into 24‐well plates with a density of 5 × 10^4^ cells per well. After 24‐hour culture, cells were incubated with EdU for 4 hours, then fixed with 4% paraformaldehyde and permeabilized by 0.5% Triton X‐100. After that, EdU staining and nuclear staining with DAPI were applied to identify proliferative cells. Finally, images were captured using confocal microscopy and the proportion of EdU‐positive cells was calculated. For plate colony formation assay, cells were seeded into 6‐well plates with a density of 1 × 10^3^ per well. After 10‐day culture, colonies were fixed with 4% paraformaldehyde, stained with 0.1% crystal violet and counted. All experiments were performed in triplicate. The transwell assay was used to assess cell migration and invasion with the transwell system (24‐well insert, pore size, 8 μm; Corning co. Ltd, Corning, New York, USA) For migration assay, 41 × 10^4^ cells in 200 μL serum‐free medium were plated into the upper chamber, and 600 μL medium containing 20% FBS was added into the lower chamber. 24 hours later, the cells that migrated to the lower side of chamber membrane were fixed with 4% paraformaldehyde, stained with 0.1% crystal violet and counted. For invasion assay, Matrigel (1:10) was polymerized in upper chamber for 45 minutes at 37°C, then 11 × 10^5^ cells in serum‐free medium were plated into the upper chamber, and 600 μL medium containing 20% FBS was added into the lower chamber. 48 hours later, the cells that invaded to the lower side of chamber membrane were fixed with 4% paraformaldehyde, stained with 0.1% crystal violet and counted.

All experiments were performed with mycoplasma‐free cells.

### 2.9 Statistic analysis

The survival analysis was assessed by KM method, log‐rank test and Cox proportional hazards regression. The expressional differences of SLC52A3 gene were analysed by unpaired t test. The statistical analysis was performed by R language (version 3.5.1). *P* values were two‐sided with a significance level of 0.05.

## 3 RESULTS

### 3.1 Clinicopathological characteristics of the research population

Our research recruited 926 GCa patients, details were included in Table 1. In them, 48% (444) patients were stage I‐II and 52% (482) were stage III‐IV. Over 88% (820) of these patients were pathologically diagnosed adenocarcinoma and with 11.4% (106) of these patients were diagnosed with mucinous adenocarcinoma. 84.2% (784) patients underwent surgery and 15.2% (140) patients did not; 73.2% (678) patients underwent chemotherapy, and 26.8% (248) patients did not receive any chemotherapies.

**TABLE 1:** Clinicopathological characteristics of FUSCC GCa patients included in the present study

Variable	Case No. (100%)
All patients	926
Age (y)	
≤59	477 (51.5)
>59	449 (48.5)
Sex	
Male	658 (71.1)
Female	268 (28.9)
Smoking	
Yes	565 (61.0)
No	361 (39.0)
Alcohol	
Yes	218 (23.5)
No	708 (76.5)
BMI	
<23	538 (58.1)
≥23	388 (41.9)
Chemotherapy	
Yes	678 (73.2)
No	248 (26.8)
Surgery	
Yes	784 (84.8)
No	140 (15.2)
Stage	
I‐II	444 (48.0)
III‐IV	482 (52.0)
Pathological type	
Adenocarcinoma	820 (88.6)
Mucus adenocarcinoma/SRCC	106 (11.4)
Differentiation grade	
Well‐moderate	214 (23.1)
Poor	712 (76.9)

Abbreviations: BMI, body mass index; FUSCC, Fudan University Shanghai Cancer Center; GCa, gastric carcinoma; SRCC, signet‐ring cell carcinoma.

### 3.2 *SLC52A3* rs13042395 C allele predict better survival in GCa patients

**FIGURE 1:**
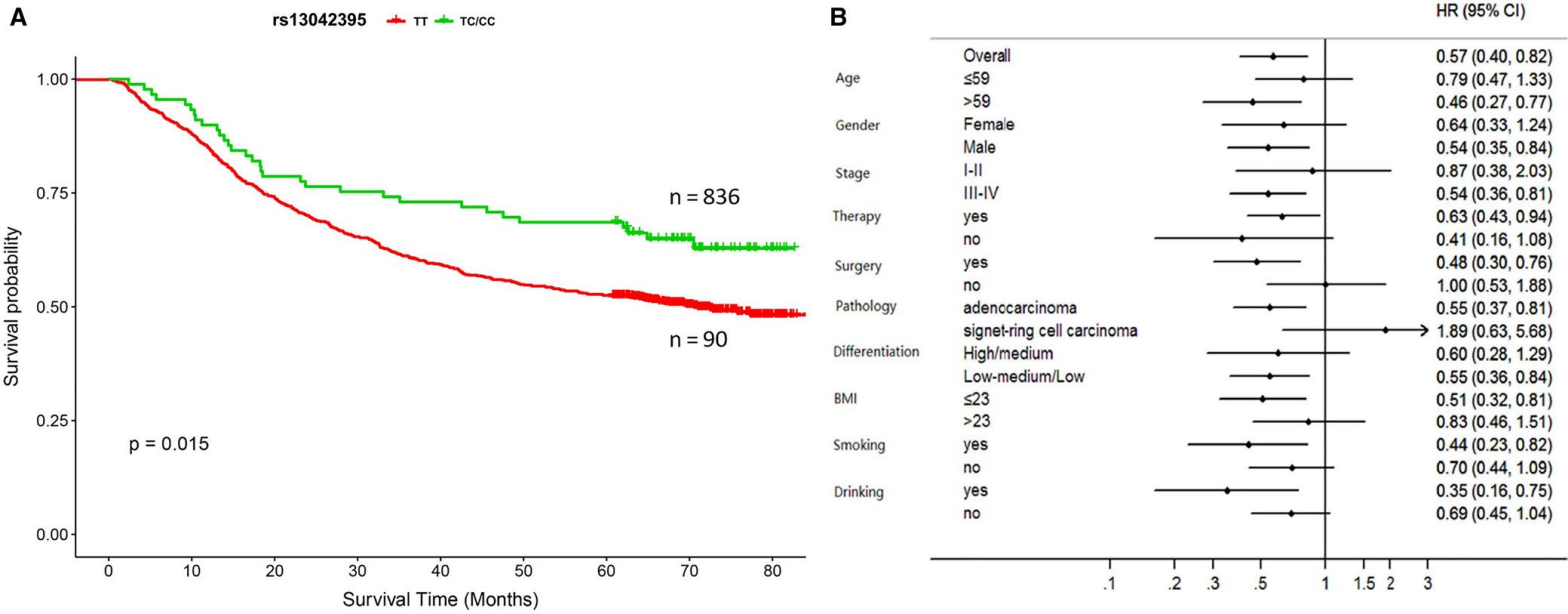
*SLC52A3* rs13042395 predicted survival of GCa patients and stratification analysis. (A) Patients of TC/CC genotype (n = 836) had a significant longer survival than the patients of TT genotype (n = 90). (B) TC/CC genotype favoured longer survival in all subgroups except the signet‐ring cell carcinoma subgroups. GCa, gastric carcinoma; BMI, body mass index

In Figure 1, we demonstrated that *SLC52A3* rs13042395 C > T change was significantly associated with OS in GCa patients with recessive model (ie TC/CC genotype versus TT genotype, log‐rank*P* = 0.015). After adjusted by clinical variables, *SLC52A3* rs13042395 was proved to be an independent prognosis factor by multivariate Cox analysis (HR = 0.57, 95%CI (0.40‐0.82), and *P* = 0.031). In the stratification analysis, *SLC52A3* rs13042395 TC/CC genotype favoured the better survival in all subgroups except the signet‐ring cell carcinoma GCa subgroup.

### 3.3 *SLC52A3* rs13042395 T allele significantly increase the *SLC52A3* expression by altering its binding affinities with *MEIS1* compared to rs13042395 C allele

**FIGURE 2:**
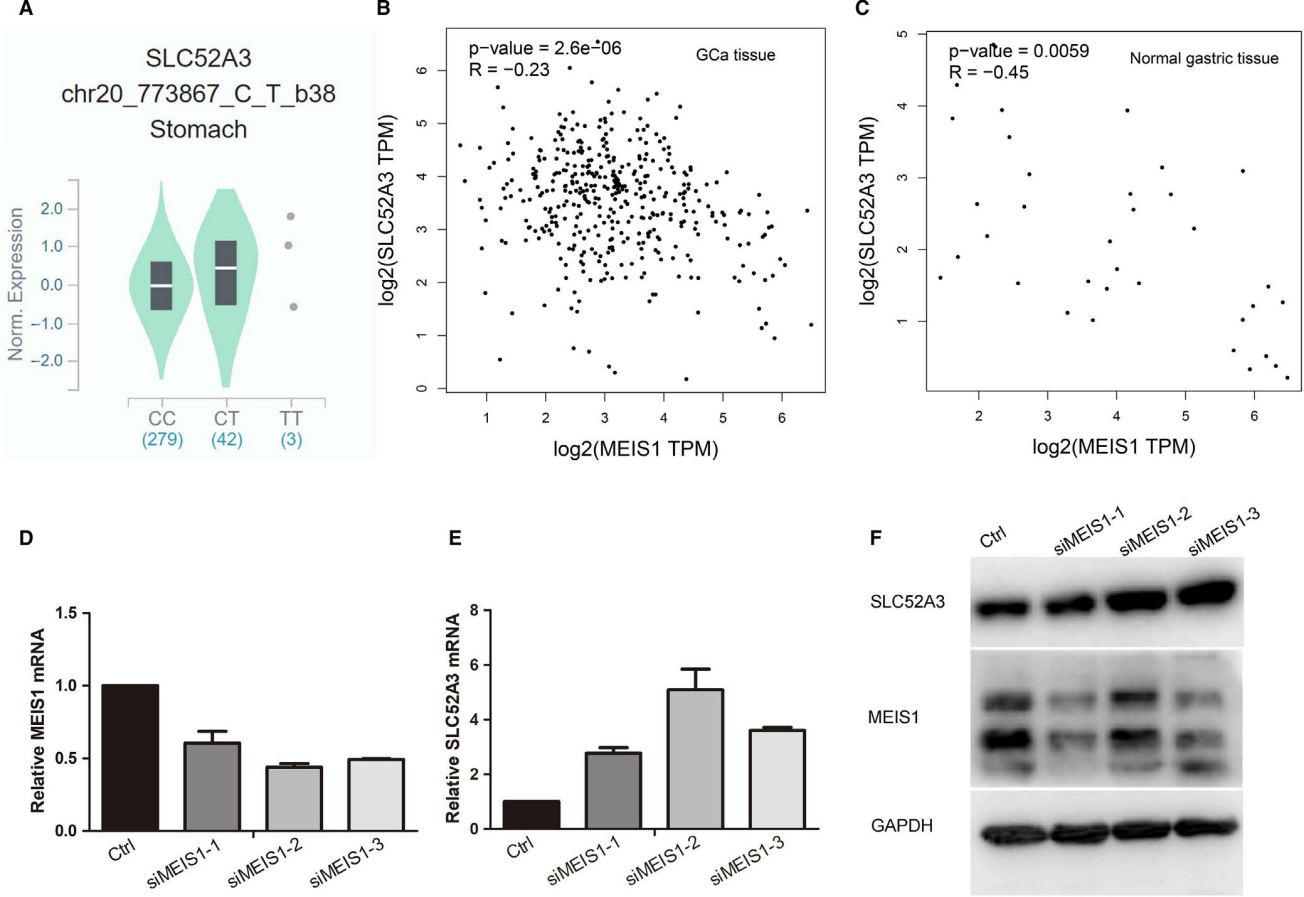
*SLC52A3* rs13042395 C > T change down‐regulated its mRNA expression via changing binding affinity with *MEIS1*. (A) In normal gastric tissue, samples with rs13042395 CT genotype had significantly higher expression level of *SLC52A3* compared to CC genotype based on GTEx database. (B‐C) In GCa tissues and normal gastric tissues, the expression of *SLC52A3* was negatively significantly associated with the *MEIS1* gene from GEPIA online tools based on TCGA database. (D‐F) Knock‐down *MEIS1* in BGC823 cells using three different siRNA led to the decreased expression of *SLC52A3* mRNA and protein. GCa, gastric carcinoma; GTEx, The Genotype‐Tissue Expression project; TCGA, The Cancer Genome Atlas

From GTEx database, we found that rs13042395 C > T change was significantly associated with higher *SLC52A3* mRNA expression level in normal gastric tissue (*P* = 0.0029) (Figure 2A).

According to the Jaspar web server, among the transcription factors which could bind to the *SLC52A3* rs13042395, the transcription factor *MEIS1* was found to have the biggest binding affinity change (specifically, the binding affinity of *MEIS1* decreased) when *SLC52A3* rs13042395 C > T change occurred (Table S1). The *MEIS1* expression was negatively associated with SLC52A3 gene expression in GCa tissue or normal gastric tissue according to the GEPIA web server which was based on TCGA data sets (Pierson correlation, *R* = −0.23,*P* < 0.00001 and *R* = −0.45, *P* < 0.01, respectively) (Figure 2B‐C). After knock‐down, the *MEIS1* using siRNA in the BGC823 cells, the *SLC52A3* mRNA and protein expression decreased significantly (Figure 2D‐F), which validated that *MEIS1* played as an inhibitory transcription factor in regulating the SLC52A3 gene.

**FIGURE 3:**
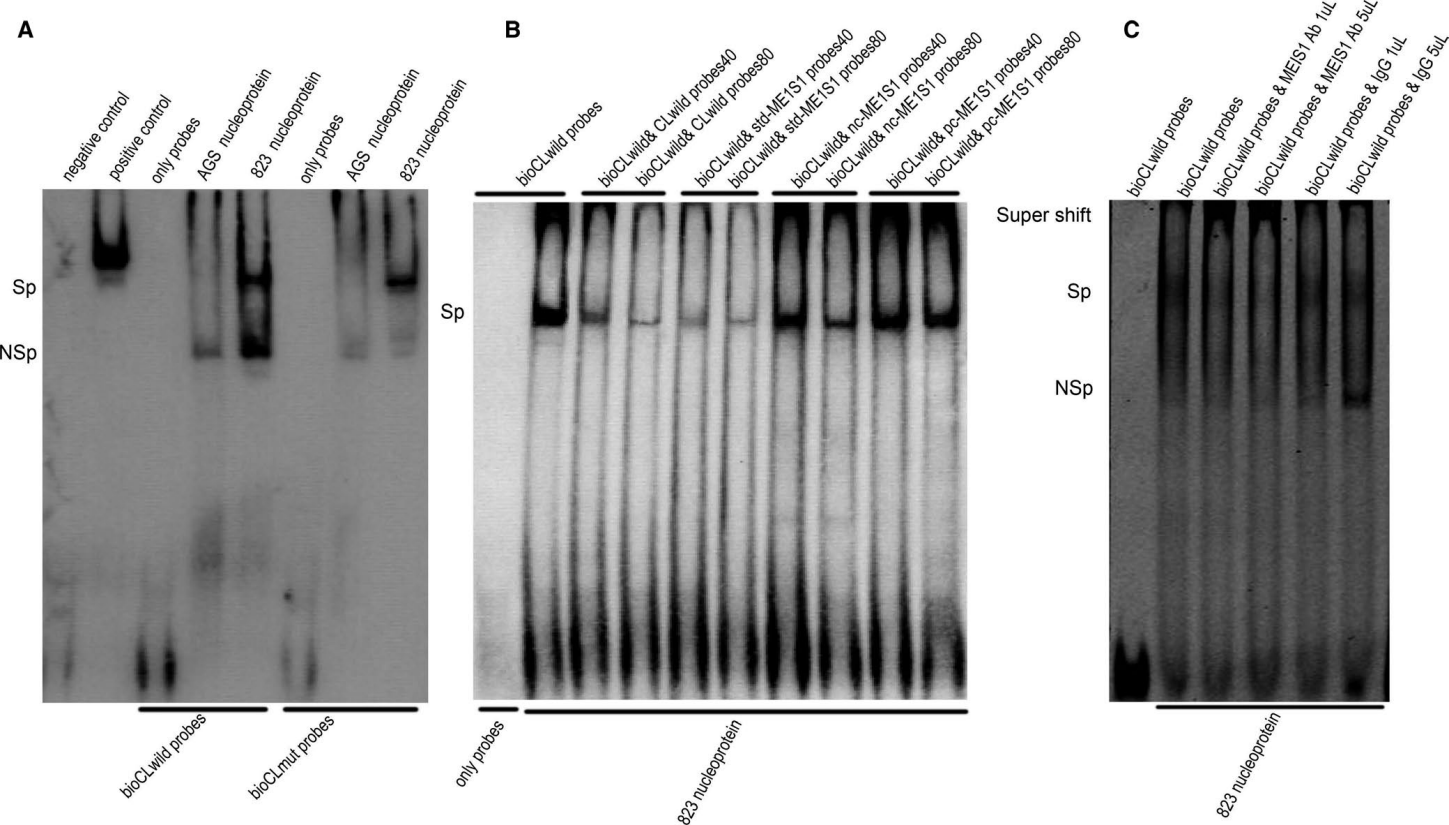
*SLC52A3* rs13042395 C allele had more firm binding affinity with *MEIS1* than the T allele. (A) *SLC52A3* rs13042395 C allele had higher binding affinities to nucleoprotein compared to T allele in BGC823 cells. (B) competitive EMSA revealed that *SLC52A3* rs13042395 C allele had higher binding affinities to *MEIS1* compared to T allele. (C) super‐shift EMSA revealed that *MEIS1* antibodies could bind to DNA‐protein complex leading to a super‐shift band. GCa, gastric carcinoma; GTEx, The Genotype‐Tissue Expression project; TCGA, The Cancer Genome Atlas; bio‐CLwild, the probe representing the rs13042395 C allele and labelled with biotin; CLwild, the probe representing the rs13042395 C allele and without labelled with biotin; bio‐CLmut, The probe representing the rs13042395 T allele and labelled with biotin; std‐MEIS1, The standard probe binding MEIS1 protein; pc‐MEIS1, The probe with modifications within the motif binding MEIS1 protein; nc‐MEIS1, the probe with modifications outside the motif binding MEIS1 protein; 40 represents 40 µmol/L; 80 represents 80 µmol/L; Ab, antibody; Sp, specific binding; NSp, non‐specific binding

Targeting sequencing showed that MGC803, AGS and BGC823 cell all carried rs13042395 CC genotype (Figure S1A). In EMSA analysis, it was revealed that the binding affinity of rs13042395 T allele probes with nucleoprotein was weaker than the rs13042395 C allele in BGC 823 cell line while this interaction of probes and nucleoprotein was not observed in AGS cell line (Figure 3A). In the competitive EMSA assay (Figure 3B), binding affinity of rs13042395 C allele probes (ie bio‐CLwild probe) with MGC823 cell nucleoprotein was attenuated after adding the rs13042395 C allele probes without labelled with biotin (ie CLwild probe), and this effect was amplified when the concentration was elevated from 40 µmol/L to 80 µmol/L. The binding affinity of bio‐CLwild probe with nucleoprotein was also attenuated by adding the standard probes for detecting the MEIS1 protein (ie std‐MEIS1 probe), and also, this effect was amplified when the concentration was elevated from 40 µmol/L to 80 µmol/L. To ensure that it was the MEIS1 transcription factor which bound to bio‐CLwild probes, we further designed the probes with modifications within the motif binding MEIS1 (ie pc‐MEIS1) and probes with modifications outside the motif binding MEIS1 (ie nc‐MEIS1). It was showed that the nc‐MEIS1 probes attenuated the binding affinity of bio‐CLwild probe with BGC823 cell nucleoprotein, and this attenuating effect was amplified when the concentration was elevated from 40 µmol/L to 80 µmol/L. However, the pc‐MEIS1 probes showed no effect on the binding affinity of bio‐CLwild probe with BGC823 cell nucleoprotein whether the concentration was 40 µmol/L or 80 µmol/L. In the super‐shift EMSA assay (Figure 3C), a super‐shifted band was demonstrated after adding *MEIS1* antibody.

Based on the above assays, it was found that the *SLC52A3* rs13042395 C allele could more firmly bind to the *MEIS1* transcription factor than the T allele, and the *MEIS1* gene served as an inhibitory transcription in regulating the *SLC52A3,* so that *SLC52A3* rs13042395 C > T change resulted in increased expression level of *SLC52A3*.

### 3.4 *SLC52A3* positively regulates malignant phenotype of gastric cancer cells

**FIGURE 4:**
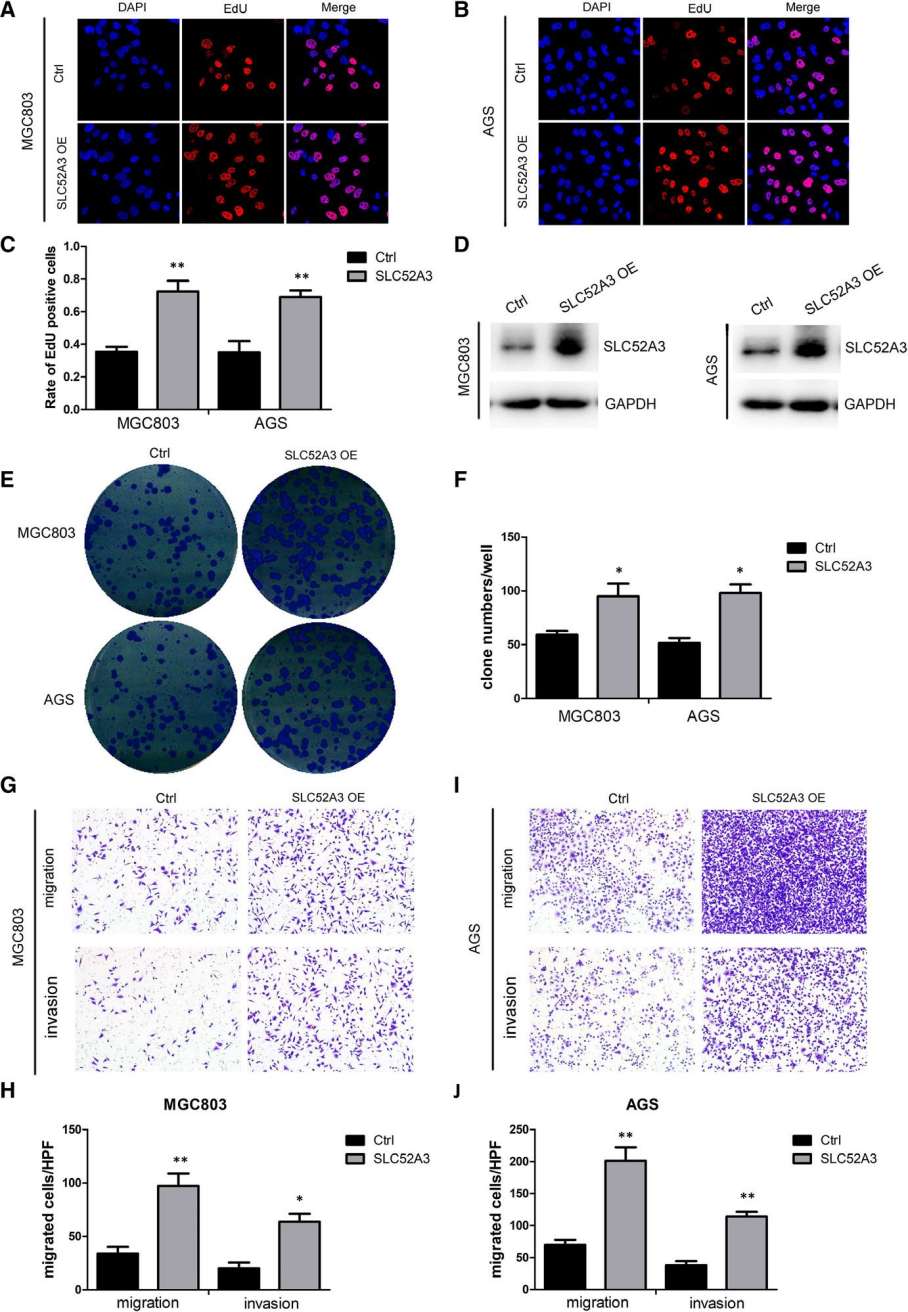
Overexpression of *SLC52A3* promoted malignant phenotype of GCa cells. *SLC52A3* was overexpressed using lentiviral vectors in MGC803 cells and AGS cells, and western blotting was used to verify the overexpression of SLC52A3 (D). The aggressive performance was assessed by EdU cell proliferation assay (A: MGC803,B: AGS, C: quantification of A and B), colony formation (E‐F), and transwell assay (G: MGC803, I: AGS, H‐J: quantification of G and I). GCa, gastric carcinoma. *P < 0.05 **P < 0.01

To explore the role of *SLC52A3* in regulating malignant phenotype of gastric cancer cells, including cell proliferation, colony formation, migration and invasion, we established stable transfected gastric cancer cell lines with MGC803 and AGS. Western blotting verified successful overexpression of *SLC52A3* in MGC803 and AGS as shown in (Figure 4D). The results of EdU cell proliferation assay and colony formation assay showed that overexpression of *SLC52A3* in both MGC803 and AGS increased rate of EdU‐positive cells and number of clones, which indicated that *SLC52A3* could promote gastric cancer cells proliferation (Figure 4A‐C, E‐F). Our results of transwell chamber assay demonstrated that overexpression of *SLC52A3* could promote gastric cancer cells migration and invasion in MGC803 and AGS (Figure 4G‐J).

### 3.5 *SLC52A3* regulates malignant phenotype of gastric cancer cells through down‐regulation of *GJA1*

**FIGURE 5:**
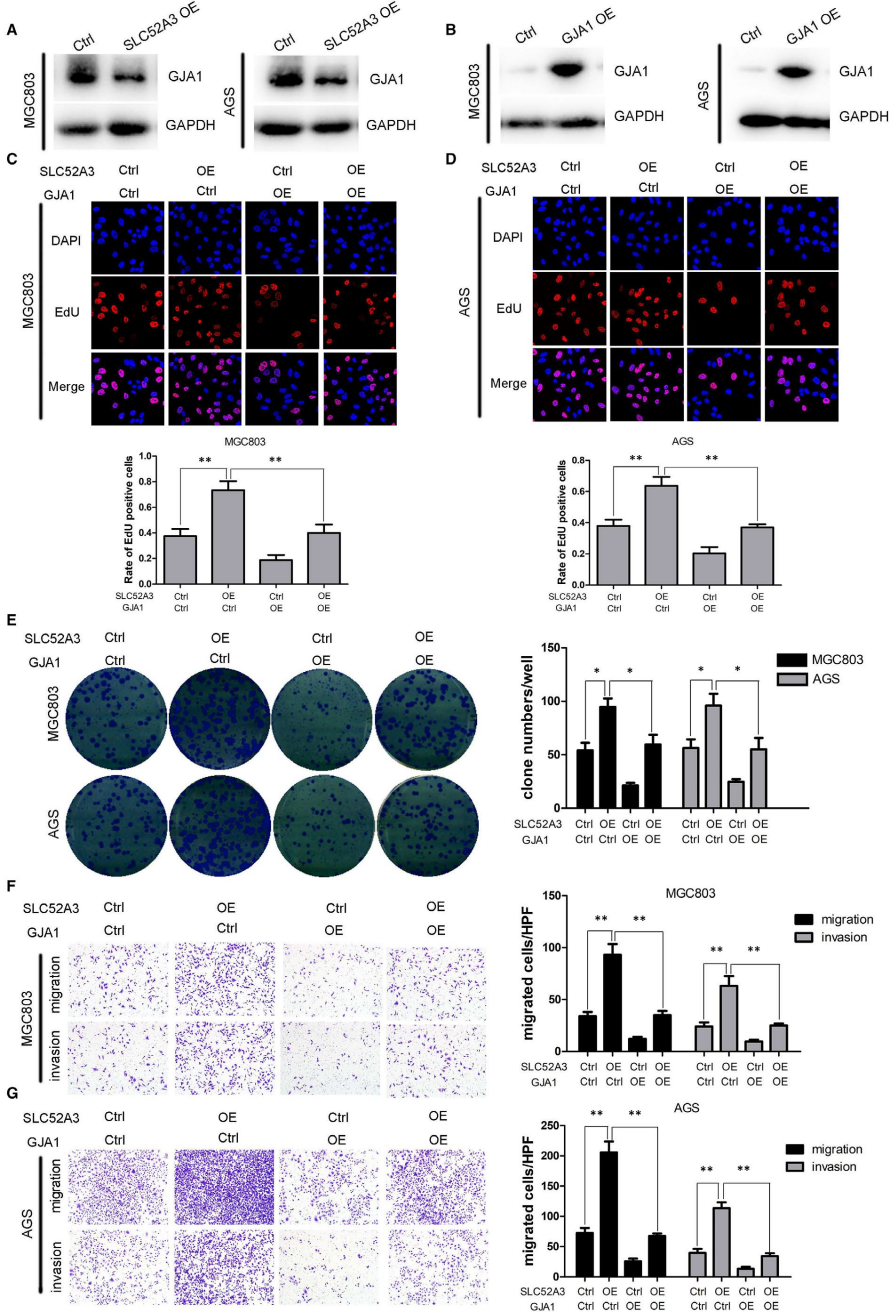
The interaction of *SLC52A3* and *GJA1* in GCa cells. Overexpression *SLC52A3* down‐regulated *GJA1* expression (A), *GJA1* overexpression by lentiviral vectors was validated by Western blotting assay (B). Overexpression of *SLC52A3* promoted cell proliferation, colony formation, migration and invasion, while *GJA1* overexpression inhibited malignant phenotype (C‐G). Co‐transfection of *SLC52A3* and *GJA1* restored proliferation, colony formation, migration and invasion of GCa cells (C‐G). GCa, gastric carcinoma. *P < 0.05, **P < 0.05

The GSEA of two GEO data sets (GSE62254 and GSE15459) and TCGA gastric cancer data set revealed that *GJA1* may be a downstream target gene for *SLC52A3* (Figure S2), we further investigated whether *SLC52A3* regulated malignant phenotype of gastric cancer cells via regulating *GJA1*. We firstly detected *GJA1* expression in *SLC52A3* overexpression gastric cancer cells and found that overexpression of *SLC52A3* resulted in decreased *GJA1* (Figure 5A). To further explore the role of *GJA1* in *SLC52A3*‐mediated malignant phenotype, we co‐transfected *GJA1* in *SLC52A3*‐expressing MGC803 and AGS gastric cancer cells and detected *GJA1* expression level by Western blotting (Figure 5B). Our results showed that overexpression of *SLC52A3* promoted cell proliferation, colony formation, migration and invasion, while *GJA1* overexpression inhibited malignant phenotype (Figure 5C‐G). Furthermore, co‐transfection of *SLC52A3* and *GJA1* restored proliferation, colony formation, migration and invasion of gastric cancer cells (Figure 5C‐G). The above results demonstrated that *SLC52A3* regulated malignant phenotype of gastric cancer cells through down‐regulation of *GJA1*. Additionally, Knockdown the *MEIS1* in BGC823 cells led to the decreased expression of *GJA1* protein (Figure S1B).

## 4 DISCUSSION

It was well established that precise prognosis prediction played an important role for treatment decision‐making for GCa, for the reason that high‐risk patients may need more aggressive interventions and low‐risk patients need avoid overtreatments.^11^ In this relatively large survival analysis in Chinese GCa patients which contained over 900 patients, we identified that *SLC52A3* rs13042395 C > T variation would independently significantly impact the OS of patients and demonstrated that *SLC52A3* expression was increased due to the different binding affinities of rs13042395 C > T change with transcriptional factors. Specifically, rs13042395 C allele had a higher binding affinity with inhibitory transcription factors *MEIS1* compared to the rs13042395 T allele so that rs13042395 T allele significantly increased the expression of *SLC52A3* compared to C allele. Further mechanism investigation found that overexpression of *SLC52A3* contributes to the increased ability of proliferation, colony formation, migration and invasion likely via down‐regulation of *GJA1* expression in vitro.

*SLC52A3* was essential for absorption of riboflavin, which was a critical component of the mitochondrial electron transport chain, and loss functional mutation of *SLC52A3* may lead to Brown‐Vialetto‐Van Laere syndrome, a rare neurological disorder characterized by bulbar palsies and sensorineural deafness.^12^ As for the cancer risk, riboflavin supplement was thought to reduce various types of cancer risks,^13^ and several cancer risk research found that *SLC52A3* SNPs was associated with oesophageal cancer risk,^14^, ^15^, ^16^ probably activated by NF‐κB p65/Rel‐B.^17^ Previous studies also found that *SLC52A3* rs13042395 C > T change could promote glioma cancer cell progression and migration in vivo and in vitro.^18^ Interestingly, researchers found that *SLC52A3* rs13042395 TT genotype favoured reduced lymph node metastasis rate and longer relapse‐free survival in oesophageal squamous cell carcinoma.^19^ Meanwhile, other researcher found that in oesophageal cancer cells, depletion of *SLC52A3* would increase p21 and p27 protein levels, decreased cyclin E1 and Cdk2 leading to cell cycle arrest at G1‐G1/S,^8^ suggested that *SLC52A3* rs13042395 C > T change played a complicated role in oesophageal squamous cell carcinoma. While in the GCa, as 90% of them were adenocarcinoma, *SLC52A3* rs13042395 C > T change played a consistent role as an oncogene both in risk or survival analysis and in our present mechanism studies.

The downstream target genes of *SLC52A3* were unclear in GCa cells so far as we know. Using GSEA analysis, we speculated that the *GJA1* may be a target gene of *SLC52A3* in GCa cells. Further research in vitro confirmed that *GJA1* gene was negative regulated by SLC52A3 gene in our study.

*GJA1*, which was enriched in the downstream of *SLC52A3* according to our research, was a member of the connexin gene family and encoded cell gap junction protein, and it was crucial for low molecular weight materials transportation from the cell to cell.^20^ Researchers already confirmed that the connexin gene family played a complicated role in tumorigenesis and progression.^21^ Recently researcher found that *GJA1* was associated in glioblastoma cancer cells apoptosis and promote the progression and invasion in breast cancer cells.^22^, ^23^ Few research about *GJA1* was conducted in GCa, one of them suggested that overexpression of *GJA1* leads to decreased ability of colony forming and invasive ability in BGC‐823 cells,^24^ which was consistent with our research results.

In conclusion, we found that *SLC52A3* rs13042395 C > T change independently predicted the survival in Chines eastern GCa patients. *SLC52A3* rs13042395 T allele had a lower binding affinity with inhibitory transcription factor, *MEIS1*, leading the up‐regulation of SLC52A3 gene, *SLC52A3* overexpression is associated with aggressive phenotype in GCa cells likely via down‐regulation of GJA1 gene. However, because our research was conducted just in one medical centre and only include Han ethnicity patients, additional multi‐centres and multi‐ethnicities studies or clinical trials as well as mechanism investigations in vivo are needed to confirm these results.

## CONFLICTS OF INTEREST

The authors have no conflicts of interest to declare.

## AUTHOR CONTRIBUTION

**Xiaofei Qu:** Conceptualization (lead); Data curation (equal); Formal analysis (equal); Investigation (equal); Methodology (equal); Software (lead); Writing‐original draft (equal). **Lei Cheng:** Conceptualization (equal); Data curation (equal); Formal analysis (equal); Funding acquisition (supporting); Investigation (equal); Methodology (equal); Writing‐review & editing (equal). **Liqin Zhao:** Formal analysis (equal); Investigation (equal); Project administration (equal); Writing‐original draft (equal). **Lixin Qiu:** Conceptualization (supporting); Data curation (supporting); Methodology (equal); Project administration (lead); Supervision (equal); Validation (equal); Writing‐review & editing (lead). **Wejian Guo:** Conceptualization (equal); Funding acquisition (lead); Project administration (lead); Resources (lead); Supervision (lead); Writing‐original draft (supporting); Writing‐review & editing (lead).

## Supporting information

Fig S1

Fig S2

Table S1

## Data Availability

The deidentification data that support the findings of this study are available on request from the corresponding author. The raw data are not publicly available due to privacy or ethical restrictions, any researcher who are interested in getting raw data are welcome to contact Fudan University institutional review board to obtain permission.
