# Correction to ‘Functional Variation of SLC52A3 rs13042395 Predicts Survival of Chinese Gastric Cancer Patients’

**DOI:** 10.1111/jcmm.70326

**Published:** 2024-12-27

X. Qu, L. Cheng, L. Zhao, L. Qiu, and W. Guo, “Functional Variation of SLC52A3 rs13042395 Predicts Survival of Chinese Gastric Cancer Patients,” Journal of Cellular and Molecular Medicine 24, no. 21 (2020): 12550–12559, https://doi.org/10.1111/jcmm.15798.

In the published article, Figure 1 was incorrectly presented, due to the incorporation of the wrong set of data. The correct Figure 1 is shown below.

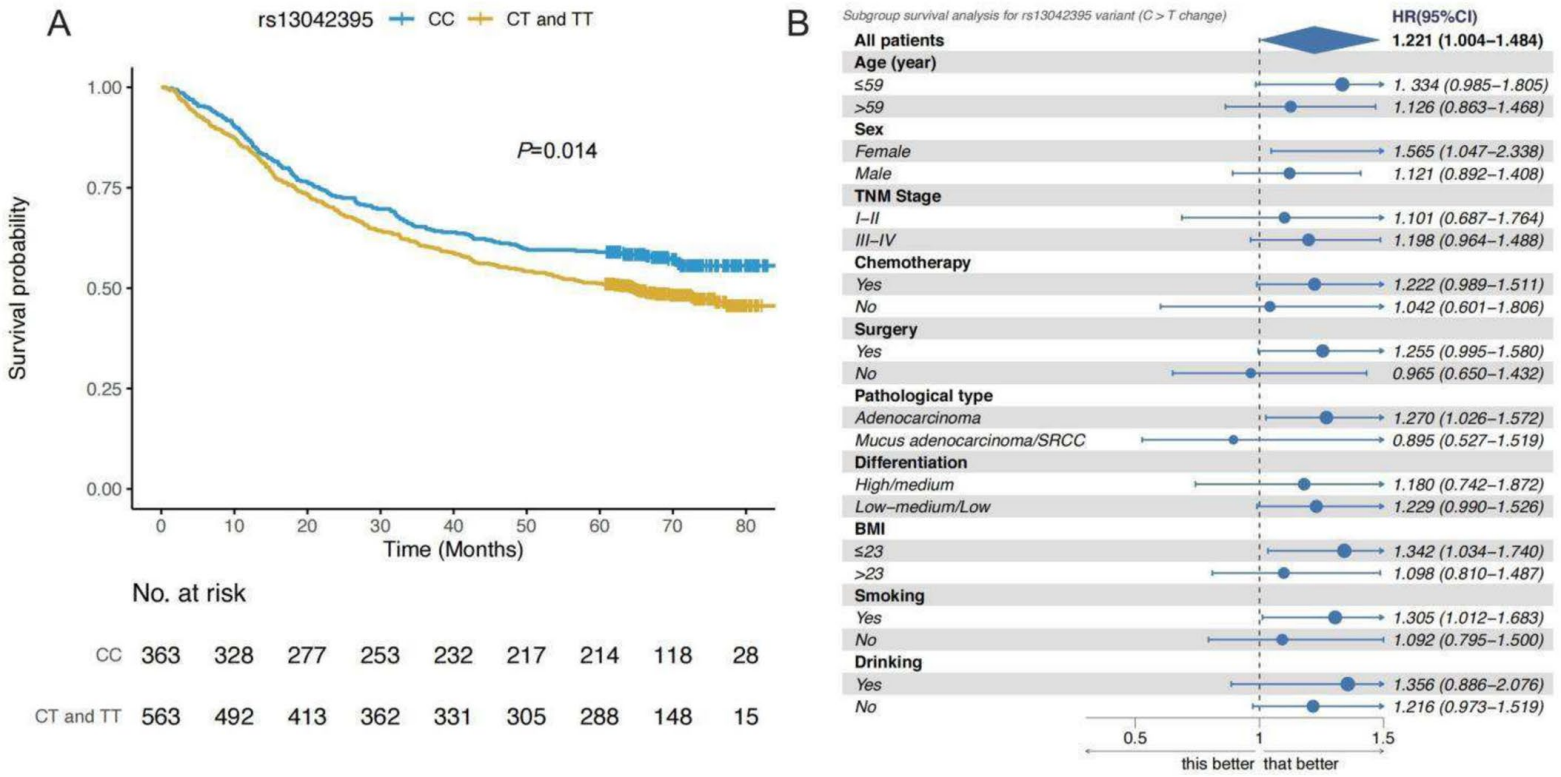

Accordingly, the legend for Figure 1 is corrected from:

‘SLC52A3 rs13042395 predicted survival of GCa patients and stratification analysis. (A) Patients of TC/CC genotype (*n* = 836) had a significant longer survival than the patients of TT genotype (*n* = 90). (B) TC/CC genotype favoured longer survival in all subgroups except the signet‐ring cell carcinoma subgroups. BMI, body mass index; GCa, gastric carcinoma’.

to:

‘SLC52A3 rs13042395 predicted survival of GCa patients and stratification analysis. (A) Patients with CT/TT genotype (*n* = 563) had a significant worse survival than the patients of CC genotype (*n* = 363). (B) The C > T change was significantly associated with worse survival in some subgroups. BMI, body mass index; GCa, gastric carcinoma’.

In ‘Abstract section’, the text:

‘SLC52A3 rs13042395 C > T variation was significantly associated with poor survival in a 926 Chinese gastric cancer (GCa) patients cohort [CC/CT genotype vs. TT genotype, HR = 0.57, 95% CI (0.40‐0.82), log‐rank *p* = 0.015]’.

is corrected to:

‘SLC52A3 rs13042395 C > T variation was significantly associated with poor survival in a 926 Chinese gastric cancer (GCa) patients cohort (HR = 1.22, 95% CI = 1.00–1.48, *p* = 0.046) after multivariate adjustment’.

Section 3.2, is corrected from:

‘In Figure 1, we demonstrated that SLC52A3 rs13042395 C > T change was significantly associated with OS in GCa patients with recessive model (i.e., TC/CC genotype vs. TT genotype, log‐rank *p* = 0.015). After adjusted by clinical variables, SLC52A3 rs13042395 was proved to be an independent prognosis factor by multivariate Cox analysis [HR = 0.57, 95% CI (0.40–0.82) and *p* = 0.031]. In the stratification analysis, SLC52A3 rs13042395 TC/CC genotype favoured the better survival in all subgroups except the signet‐ring cell carcinoma GCa subgroup’.

to:

‘In Figure 1, we demonstrated that SLC52A3 rs13042395 C > T change was significantly associated with OS in GCa patients with dominant model (CC vs. CT/TT genotypes, log‐rank *p* = 0.014)’. After adjusted by clinical variables, SLC52A3 rs13042395 C > T change was proved to be an independent predictor for worse survival by multivariate Cox analysis (HR = 1.22, 95% CI = 1.00–1.48, *p* = 0.046). In the stratification analysis, ‘SLC52A3 rs13042395 C > T change was significantly associated with poor survival in some subgroups, including female patients, and those with gastric adenocarcinoma, a smoking history or BMI ≤ 23’.

In the paragraph 3 of section 3.3, there was a typographical error ‘MGC823’ which should be corrected as ‘BGC823’.

The sequence of the primers for Sanger sequencing in section 2.4 was missing in the published article, and is included in this correction:

F: GAGAAGCTGAGAGAGGCTC; R: CACTGGGGGAGAGGACAAGC.

The authors confirm that this correction does not change the results and conclusions of the article, and apologise for the inconvenience that these errors may have caused.

