# Supplementary figures and images for: Functional variation of SLC52A3 rs13042395 predicts survival of Chinese gastric cancer patients
Source: J Cell Mol Med. 2020 Sep 5;24(21):12550–9. doi: 10.1111/jcmm.15798 (PMC7686988; doi:10.1111/jcmm.15798)

**A**

rs13042395 flank sequence ACCAGGGCCAGTGCACCGT[C/T]ATTGTGTGGGCTGGGCCAT

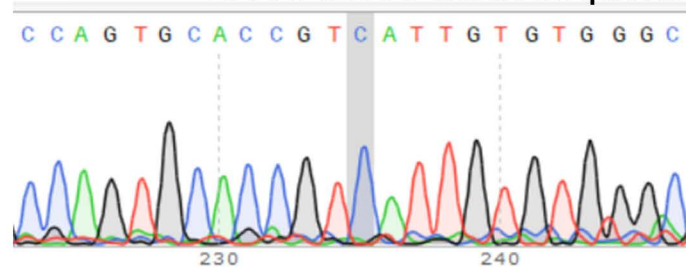

MGC803

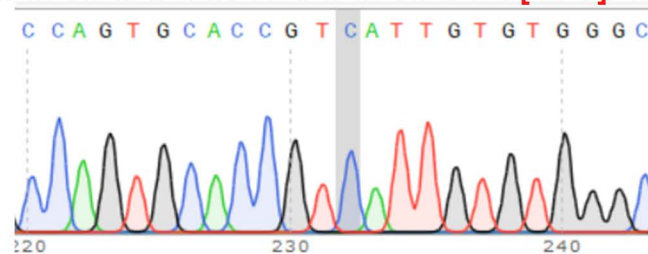

BCG823

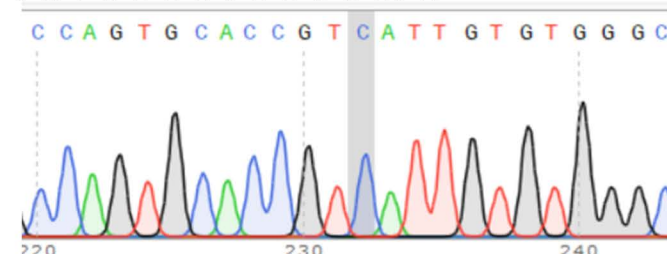

AGS

**B**

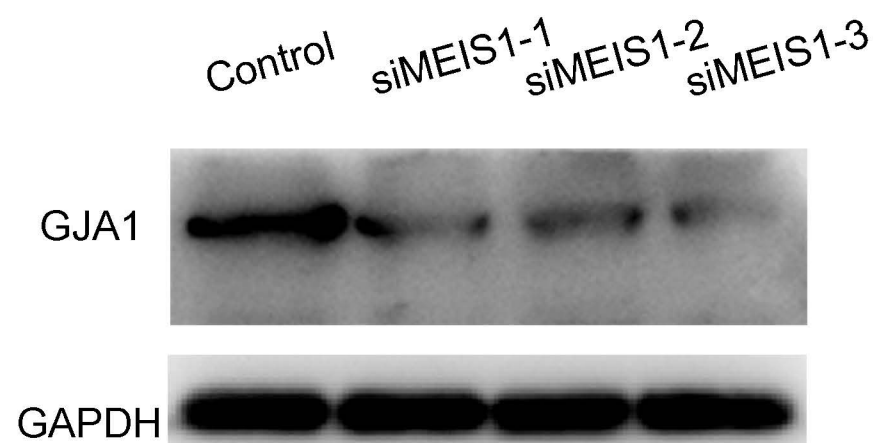

Supplement: Supplementary file 1 — Fig S1 [file JCMM-24-12550-s001.pdf]
