# Supplementary material for: Functional variation of SLC52A3 rs13042395 predicts survival of Chinese gastric cancer patients
Source: J Cell Mol Med. 2020 Sep 5;24(21):12550–9. doi: 10.1111/jcmm.15798 (PMC7686988; doi:10.1111/jcmm.15798)
Supplement: Supplementary file 2 — Fig S2 [file JCMM-24-12550-s002.pdf]

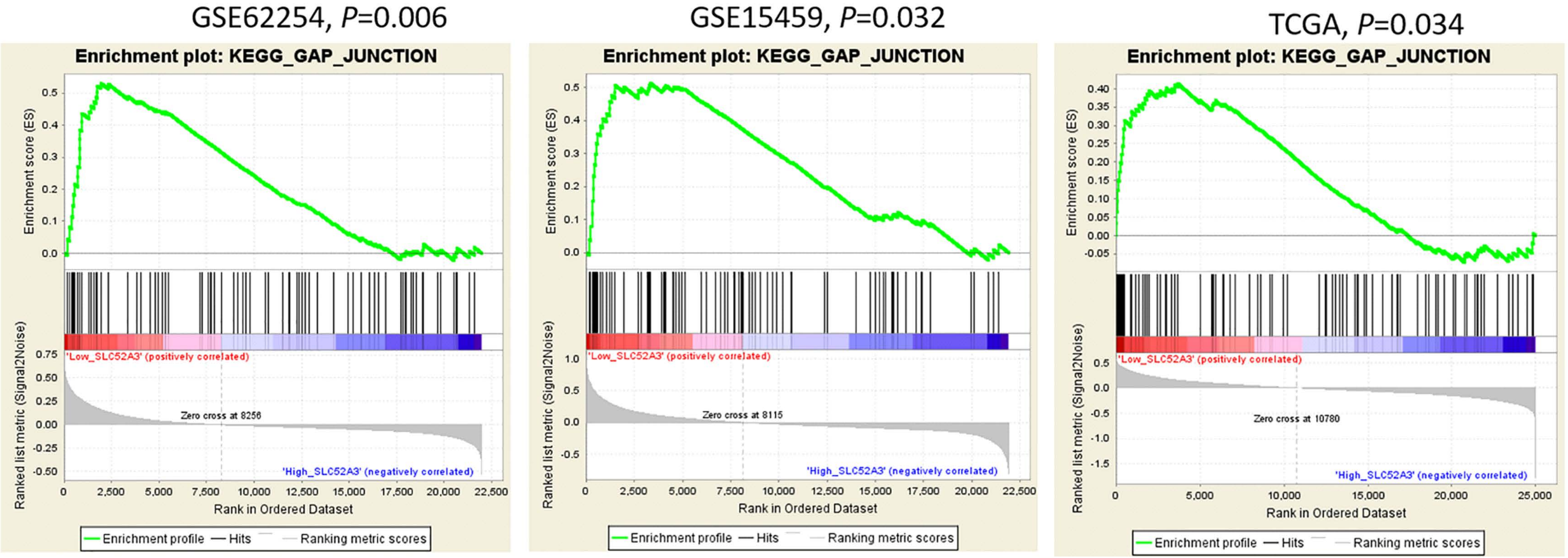

GSE62254, P=0.006
Enrichment plot: KEGG_GAP_JUNCTION
Enrichment score (ES)
'Low_SLC52A3' (positively correlated)
Zero cross at 8256
'High_SLC52A3' (negatively correlated)
Ranked list metric (Signal2Noise)
Rank in Ordered Dataset
Enrichment profile
Hits
Ranking metric scores
GSE15459, P=0.032
Enrichment plot: KEGG_GAP_JUNCTION
Enrichment score (ES)
'Low_SLC52A3' (positively correlated)
Zero cross at 8115
'High_SLC52A3' (negatively correlated)
Ranked list metric (Signal2Noise)
Rank in Ordered Dataset
Enrichment profile
Hits
Ranking metric scores
TCGA, P=0.034
Enrichment plot: KEGG_GAP_JUNCTION
Enrichment score (ES)
'Low_SLC52A3' (positively correlated)
Zero cross at 10780
'High_SLC52A3' (negatively correlated)
Ranked list metric (Signal2Noise)
Rank in Ordered Dataset
Enrichment profile
Hits
Ranking metric scores
