# Supplementary material for: Functional variation of SLC52A3 rs13042395 predicts survival of Chinese gastric cancer patients
Source: J Cell Mol Med. 2020 Sep 5;24(21):12550–9. doi: 10.1111/jcmm.15798 (PMC7686988; doi:10.1111/jcmm.15798)
Supplement: Supplementary file 3 — Table S1 [file JCMM-24-12550-s003.doc]

| **Supplemental Table1**. SLC52A3 rs13042395 C>T change resulted decreased binding affinity with inhibitory transcription factor MEIS1 from the Jaspar web server | | | | | | | | | |
| --- | --- | --- | --- | --- | --- | --- | --- | --- | --- |
| rs13042395 C allele sequence flanked by 29 bases | | | | | |  |  |  |  |
| TGGGGTTCTGACCAGGGCCAGTGCACCGT**C**ATTGTGTGGGCTGGGCCATCTCCTCCAGG | | | | | | | | | |
| Matrix ID | Name | Score | Relative score | Sequence ID | Start | End | Strand | Predicted sequence | |
| MA0498.2 | MEIS1 | 7.23429 | 0.933946191001 | seq1 | 26 | 32 | - | ATGACGG | |
|  |  |  |  |  |  |  |  |  |  |
| rs13042395 T allele sequence flanked by 29 bases | | | | | |  |  |  |  |
| TGGGGTTCTGACCAGGGCCAGTGCACCGT**T**ATTGTGTGGGCTGGGCCATCTCCTCCAGG | | | | | | | | | |
| Matrix ID | Name | Score | Relative score | Sequence ID | Start | End | Strand | Predicted sequence | |
| MA0498.2 | MEIS1 | -2.8376 | 0.720652793305 | seq1 | 26 | 32 | - | ATAACGG | |

The red and bold character was the mutated SNP
